# Role of cell type and synaptic connections during functionally relevant network states *in vitro*

**DOI:** 10.1186/1471-2202-14-S1-P311

**Published:** 2013-07-08

**Authors:** Antonio Pazienti, Alberto Bacci

**Affiliations:** 1 European Brain Research Institute, Rome, Italy; 2 Institut du Cerveau et de la Moelle épinière, Paris, France

## 

Cortical neurons are embedded in networks, whose activity has distinct features depending on the brain state and on the involved cell types. Two distinct types of functionally relevant activities are asynchronous firing containing spike correlations and temporally-structured, oscillatory activity. In the neocortex, network synchronization is achieved by the tight control of principal pyramidal, excitatory neuron (PN) firing by fast-spiking (FS), parvalbumin-positive inhibitory neurons. However, how different neuron types process and convey information in different cortical states is not completely understood. We simultaneously recorded from pairs of neurons in acute mouse cortical slices. Using dynamic clamp, we combined computer modeling with experimental electrophysiology and we studied how cortical neurons compute and integrate diverse sequences of synaptic inputs (both excitatory and inhibitory) into specific spike-train outputs. In particular, we used three types of inputs: *i) *asynchronous, correlated spike trains in the form of post-synaptic currents, as originating from a network of pre-synaptic randomly connected neurons; *ii) *sinusoidal currents of various frequencies (ranging from the delta to the high-gamma bands); *iii) *realistic excitatory and inhibitory oscillating currents in the gamma frequency band. For all these stimuli, we also tested the effect of an *in vivo*-like synaptic high-conductance state on neuronal response [1].

For asynchronous input containing correlations, we measured output spike correlations between pairs of two PNs, two FS cells, or one PN and one FS cell at different degrees of correlation. Our results show that inhibition desynchronizes the output of PNs, preventing supra-linear correlation [2]. Moreover, this occurred also when neurons were injected with additional *in vivo*-like synaptic conductances. FS cells showed similar sensitivity to inhibition, but with a much stronger ability to correlate their output.

When sinusoidal input was injected, both PNs and FS cells followed the stimuli, with FS cells capable of firing reliably in response to high-frequency (>100 Hz) oscillatory input. Interestingly, in the presence of *in vivo*-like synaptic activity, PNs significantly increased their ability of following intracellular injections of sinusoidal input across a broader range of frequencies.

Finally, we injected realistic gamma input, by simulating and injecting runs of EPSCs and IPSCs occurring in temporally structured manner [3]. In addition, neurons were connected by artificial, albeit realistic, unitary excitatory, inhibitory and self-inhibiting synapses (autapses), thus allowing the investigation of local network effects in eliciting and maintaining oscillations. Our preliminary results show that, in pairs of FS cells, the presence of bidirectional inhibitory synapses strongly decreased the strength of output oscillations, with neurons alternatively silencing each other. Interestingly, additional autaptic GABAergic conductances balanced the network, allowing reemergence of oscillations.

Overall, our results deepen our understanding of the features and the processing capabilities of excitatory and inhibitory networks of neurons in the presence of functionally relevant brain activities.
